# Emerging Developments in Separation Techniques and Analysis of Chiral Pharmaceuticals

**DOI:** 10.3390/molecules28176175

**Published:** 2023-08-22

**Authors:** Sulaiman Al-Sulaimi, Reveka Kushwah, Mohammed Abdullah Alsibani, Atef El Jery, Moutaz Aldrdery, Ghulam Abbas Ashraf

**Affiliations:** 1 Department of Biological Science and Chemistry, University of Nizwa, Nizwa 611, Oman; s.salimi@unizwa.edu.om (S.A.-S.); k.revekakushwah@gmail.com (R.K.); sibanimm@unizwa.edu.om (M.A.A.); 2 Department of Chemical Engineering, College of Engineering, King Khalid University, Abha 61411, Saudi Arabia; 3 College of Environment, Hohai University, Nanjing 210098, China

**Keywords:** chiral separation, capillary electrophoresis, chiral selectors, chiral drugs, HPLC, NSAID, microfluidics system, monolithic column

## Abstract

Chiral separation, the process of isolating enantiomers from a racemic mixture, holds paramount importance in diverse scientific disciplines. Using chiral separation methods like chromatography and electrophoresis, enantiomers can be isolated and characterized. This study emphasizes the significance of chiral separation in drug development, quality control, environmental analysis, and chemical synthesis, facilitating improved therapeutic outcomes, regulatory compliance, and enhanced industrial processes. Capillary electrophoresis (CE) has emerged as a powerful technique for the analysis of chiral drugs. This review also highlights the significance of CE in chiral drug analysis, emphasizing its high separation efficiency, rapid analysis times, and compatibility with other detection techniques. High-performance liquid chromatography (HPLC) has become a vital technique for chiral drugs analysis. Through the utilization of a chiral stationary phase, HPLC separates enantiomers based on their differential interactions, allowing for the quantification of individual enantiomeric concentrations. This study also emphasizes the significance of HPLC in chiral drug analysis, highlighting its excellent resolution, sensitivity, and applicability. The resolution and enantiomeric analysis of nonsteroidal anti-inflammatory drugs (NSAIDs) hold great importance due to their chiral nature and potential variations in pharmacological effects. Several studies have emphasized the significance of resolving and analyzing the enantiomers of NSAIDs. Enantiomeric analysis provides critical insights into the pharmacokinetics, pharmacodynamics, and potential interactions of NSAIDs, aiding in drug design, optimization, and personalized medicine for improved therapeutic outcomes and patient safety. Microfluidics systems have revolutionized chiral separation, offering miniaturization, precise fluid control, and high throughput. Integration of microscale channels and techniques provides a promising platform for on-chip chiral analysis in pharmaceuticals and analytical chemistry. Their applications in techniques such as high-performance liquid chromatography (HPLC) and capillary electrochromatography (CEC) offer improved resolution and faster analysis times, making them valuable tools for enantiomeric analysis in pharmaceutical, environmental, and biomedical research.

## 1. Introduction

**Figure 1:**
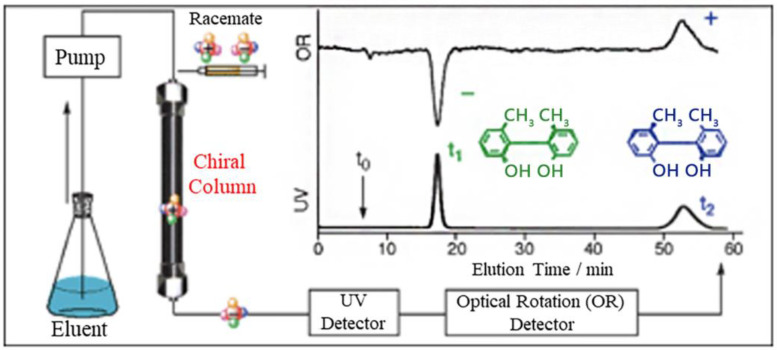
Chiral HPLC for effective enantiomer separation (Copyright “Chemical Society Reviews”, 2008) [3].

The investigation of medication pharmacokinetics, peptide synthesis, and asymmetric synthesis in organic chemistry are just a few areas where the enantiomer separation of chiral substances is crucial [1]. Typically, chiral separation procedures can be divided into three groups: (i) separations utilizing chiral selectors like cyclodextrin and crown ethers in the operating buffer or mobile phase of high-performance liquid chromatography (HPLC) or capillary electrophoresis (CE), (ii) chiral stationary-phase separations using materials like cellulose (3,5-dimethylphenyl- carbamate) or amylose, and (iii) enantioseparation based on the production of diastereomers using chiral derivatization methods such as 4-nitro-7-(3-aminopyrrolidin-1-yl)2,1,3-benzoxadiazole, 4-(*N*,*N*-dimethylaminosulphonyl)-7-(3-aminopyrrolidin1-yl)-1,3-benzoxadiazole, and 4-(aminosulphonyl)-7-(3-aminopyrrolidin-l-yl)-2,1,3-benzoxadiazole. Each of these methods has advantages and disadvantages [2]. Derivatization reactions and separations using HPLC are well recognized to present a number of difficulties. One of these is that precolumn derivatization with chemicals has a slow reaction rate, uses a lot of reagents and solvents, and takes a long time to complete, making routine HPLC analysis a costly process. Solvents and many other reagents have seen recent price increases. Novel analytical systems that will speed up reactions and reduce the need for pricey reagents and solvents are therefore necessary. Figure 1 describes the steps involved in chiral separation [3].

### 1.1. Importance of Chiral Separation

The study of enantiomers is crucial in pharmaceutical design and deployment since many pharmaceutically active compounds contain at least one chiral center. This is because, although one enantiomer might have the desired pharmacological effect, the other could be of a lower potency or even toxic. Each stereoisomer of a racemic medication made up of one or more pairs of stereoisomers may react differently pharmacologically when it interacts with a receptor or an enzyme. When creating pharmaceuticals with stereogenic carbon atoms in their structures, the U.S. Food and Drug Administration (FDA) mandates the synthesis of single individual stereoisomers rather than the use of racemic mixes [4]. Because the two enantiomers’ pharmacological activity and metabolism differ often, it is necessary to determine each one separately using proper analytical techniques [5]. Because of the body’s incredible chiral selectivity, each racemic drug will interact with it differently, and each enantiomer will be metabolized through a distinct metabolic pathway, leading to a different pharmacological function. As a result, one isomer may result in the desired therapeutic results, whilst the other may have no effect or, in the worst-case scenario, result in negative effects [6]. Therefore, due to variations in the biological activities and pharmacokinetic characteristics of pharmacological enantiomers, the enantiomeric separation of chiral medicines has emerged as a crucial topic in analytical clinical chemistry in recent years [7]. Due to challenges in stereoselective production and purification, many medicinal compounds with chiral centers are therapeutically delivered as racemic mixtures. In a stereospecific biological environment, such as the human body, differences in pharmacological qualities, pharmacokinetic disposition, and metabolism rates are frequently seen. In a symmetric environment, the isomers have almost equal physical and chemical properties [8]. Techniques like gas chromatography (GC), thin-layer chromatography (TLC), high-performance liquid chromatography (HPLC), super-critical fluid chromatography (SFC), and CE have been employed as analytical approaches so far for the analysis of chiral substances [6]. Chiral separation is important in many fields of science and industry and has significant effects on chemistry, pharmacy, biochemistry, and medicine. Enantiomers are produced as a result of the chirality property, which is related to the asymmetry in molecular structures and results in enantiomers that interact differently with biological systems. In many applications, the ability to separate and evaluate these enantiomers is crucial.

Chiral separation techniques are essential for producing safe and effective pharmaceuticals during the drug development process. Enantiomers of chiral medicines can have different biological activity and possibly different therapeutic effects. Enantiomeric purity must therefore be accurately determined in order to optimize medication design, comprehend pharmacokinetics and pharmacodynamics, and weigh potential side effects. Individual enantiomers can be isolated and thoroughly characterized using methods including chromatography, electrophoresis, and crystallization, making it easier to evaluate their biological activity [9].

Chiral separation techniques are also very important in analytical chemistry, where the enantiomeric excess measurement is essential for quality assurance and legal compliance. The integrity and consistency of pharmaceutical formulations are guaranteed by the capacity to precisely quantify the enantiomeric content of a sample. Moreover, chiral separation is important for monitoring and removing chiral contaminants in environmental analysis. Various chiral contaminants may have varying toxicities and rates of breakdown, necessitating a customized assessment and elimination strategy [10].

In order to produce chiral intermediates and fine compounds, chiral separation techniques are extremely advantageous to the industrial sector. Enantiomer separation enables the creation of valuable compounds with particular stereochemical characteristics, improving the efficiency and selectivity of chemical reactions. Chiral separation technology enables the manufacture of enantiomerically pure substances, which can have major practical and economic ramifications. This contributes to enhanced production methods [11].

Effective techniques for chiral drug analysis include capillary electrophoresis (CE) and high-performance liquid chromatography (HPLC). The advantages of CE include a high separation efficiency, short turnaround times for analyses, minimal sample consumption, and excellent reproducibility. It allows for the investigation of chiral drug metabolism, examination of potential stereoselective toxicity, and interaction with target receptors [12]. Contrarily, HPLC offers a superior resolution, high sensitivity, broad applicability, and compatibility with a range of detection techniques. It is crucial for checking the validity, pharmacokinetics, and pharmacodynamics of chiral drugs at various stages of the drug development process [13].

Additionally, the development of monolithic columns and microfluidics technologies has opened up new possibilities for chiral separation. Microfluidics devices include advantages including downsizing, high throughput, and fine fluid dynamic control, allowing for effective and quick enantiomer resolution. For improvements in pharmaceuticals, analytical chemistry, and biological research, on-chip chiral analysis’ integration of microscale channels, separation methods, and detection methods appears promising [14]. Monolithic columns are also useful instruments in chiral separation, offering better resolution, quicker analysis times, and higher sample throughput thanks to their distinctive porous structure, high permeability, and improved removal efficiency [15].

Techniques for chiral separation are extremely important in many fields of science and industry. A greater comprehension of the characteristics and effects of chiral entities is made possible by the capacity to separate and isolate them, which improves treatment outcomes, environmental protection, and industrial operations. Continuous improvements in chiral separation technology, such as those in CE and HPLC, as well as the incorporation of microfluidics systems and monolithic columns, increase their potential for enantiomeric analysis and boost drug development, quality assurance, and patient care [16,17].

### 1.2. CE for Chiral Drugs Analysis 

The chiral separation of pharmaceuticals that are employed as racemic mixes or the enantiomeric purity testing of single-isomer entities, chiral discrimination, researching chiral interactions, and measuring the binding constants of enantiomers have all been successfully accomplished using CE [18]. CE is the perfect method for chiral analysis due to a number of benefits. Baseline separation is made achievable even at relatively low selectivities thanks to the excellent removal efficiency. Additionally, the baseline resolution of enantiomer pairings with apparent selectivity values less than 1.01 is made achievable by the CE efficiency of several hundred thousand plates. Additionally, the flat U-profile of the flow and the extremely low resistance of the mass-transfer processes are the sources of the high efficiency. Faster mass transfer, rapid flow, and short columns enable quick analysis. 

Additionally, due to high efficiency, independent peak measurement, and the use of high-sensitivity detectors, trace enantiomer impurities below 0.1 percent can commonly be identified. Even traces of enantiomers might be found at 0.02 percent levels. Additionally, compared to other separation techniques, CE offers far greater capability for the small first migration order. Because the tailing of the primary peak does not affect the accurate quantification of the minor component, this migration sequence results in a more sensitive identification of trace impurities. Any selector that has been implemented in the field of HPLC or GC can likewise be used in CE [19].

Since the chiral selector is typically present in the buffer solution when utilizing CE for enantiomeric separations, it is simple to switch selectors. As a result, the CE technique can be used to quickly screen the performance of a variety of chiral selectors. The process is less expensive than, say, chiral HPLC since only minimal amounts of a selector are required due to the system’s size. Consequently, screening a variety of selections can be performed quite cheaply [20]. Additionally, the buffers can be of the water-based, organic, or polar organic types, each of which is anticipated to have unique interaction characteristics. To maximize separations, CE offers very adjustable analysis conditions. Additionally, by using well-developed optimization techniques, the composition of electrolytes (concentration and kind of selections, ionic strength, pH, etc.) may be adjusted rapidly and easily. A single living cell can be used for chiral analysis utilizing CE, which only needs very little amounts of material [21]. The use of nonaqueous electrolyte solutions in CE is gaining popularity despite the fact that the majority of enantioresolution techniques have been developed in aqueous background electrolytes. Because organic solvents have diverse physicochemical characteristics, nonaqueous CE may offer a range of selectivities [22]. One or more chiral selectors must be used in CE to separate chiral substances. By adding cyclodextrins (CDs) to the running buffer, the direct enantiomeric separation method used in CE is the most popular [23]. The cyclic oligosaccharides known as CDs include 6, 7, or 8 glucose units, linked by α-1,4 bonds and include α-, β-, and γ-cyclodextrins [24]. However, because the parent CDs are neutral substances, it is obviously impossible to use them with neutral guests. But, also with charged visitors, as there is no difference between the complex’s electric charge and that of the free ligand [25]. The selectivity of the enantiomer’s separation can be easily improved by modifying native CDs through chemical reactions to produce CD derivatives. Nowadays, CE uses a large variety of CD compounds for chiral analysis including uncharged methylated-, hydroxyethylated-, hydroxypropylated-, acetylated-CDs and the charged methylamino-, sulfobutylether-, carboxymethylated-, sulphated-, phosphate-CDs, etc. [26,27]. Numerous occurrences form the foundation of CDs’ extensive chiral recognition capabilities. Five chiral centers make up the selectors, one for each glucose unit. The chair structure contains each glucose unit. Due to the truncated cone morphologies of the molecules, the shapes of the glucose units do not repeat from unit to unit. So, β-CD has 35 diverse chiral recognition places. The reason CDs have substantially wider chiral recognition spectra than linear oligoglucosides, where all glucose units have the same shape, is due to their twisted form. Additionally, CDs have the ability to alter their form to interact with analytes, resulting in what are known as “induced-fit” interactions. The chiral selectivity spectrum of CDs is further widened by the induced-fit interactions; specifically, the analyte-driven deformation of a CD can lead to a three-point contact with one of the enantiomers, which is not conceivable with a selector’s steady-state shape [28].

Conversely, compared to other stiff selectors, the flexible structure of CDs only gives moderate selectivity values (e.g., imprinted cellulose). The application scope for the ionizable CDs is further expanded by the fact that they can alter their chiral recognition properties in accordance with their ionization states. The application of CDs in water-based, polar organic, and polar buffers can result in a variety of selectivity. For chiral recognition, for instance, the nonaqueous system provides ion-pair critical interactions [13]. Aside from being commercially available in a variety of native and derivatized forms, cyclodextrins (CDs) have also been demonstrated to be broad-spectrum chiral selectors due to their UV transparency, durability, and broad enantiorecognition capabilities. Chemical transformation of the indigenous α-, β-, and γ-CDs led to a substantial development in the physicochemical properties and chiral recognition capacities [24]. The enantioseparation of some chemicals using single cyclodextrin systems has occasionally proven challenging. As a result, dual systems combining cyclodextrins have been developed to improve selectivity and resolution. Because the two cyclodextrins’ processes for complexing with the analyte enantiomers differ in terms of complexation stability, chiral recognition pattern, and impact on analyte mobility, this combination may produce results with a higher resolution [29].

#### Chiral Drug Separations for CE

Researchers used trimethyl-β cyclodextrin (TM-β-CD) as a chiral selection for the enantiomer analysis of some 2-arylpropionic acid derivatives by CE. These enantiomers’ pharmacokinetic, bioavailability, and optical purity investigations can use their methods because they were stereospecific, sufficiently accurate, and suited for those tasks. Ketoprofen (KTP) enantiomers were highly resolved (R_s_: 3.77–4.32) [21,30,31,32], while ibuprofen (IBP) enantiomers and their major metabolites carboxy-ibuprofen/hydroxy-ibuprofen in human urine and plasma have lower enantioresolutions than KTP (1.04–2.26). The findings have validated the chiral reagent’s outstanding qualities for both the IBP enantiomers and their metabolites, which differ in polarity from the parent medication [21,33]. The IBP, KTP, fenoprofen (FNP), and naproxen (NPX) resolution in human serum were within the range (1.88–3.70). However, the resolution of indoprofen (IND) enantiomers was weak (R_s_: 1.5) [34].

The single-isomer polyanionic cyclodextrin (CD) derivative heptakis-6-sulfato-β-cyclodextrin (HS-β-CD) used as a chiral additive for the enantioseparation of nonsteroidal anti-inflammatory drugs (NSAIDs), such as IBP, FNP, flurbiprofen (FL), and KTP, in CE was only poorly resolved (R_s_: 1.3–1.4) with HS-β-CD alone [5]. But the usage of HS-β-CD in mixture with the neutral CD derivative, TM-β-CD, caused a high enantioselectivity (R_s_: 2.9–6.1) near these compounds. Also, Magnusson et al., exemplified the setback of enantiomeric elution instruction and discovered enantiomeric impurity in CE for IBP. Poor IBP resolution with HS-β-CD (R_s_: 0.7) was well resolved (R_s_: 3.3) with dual cyclodextrin (TM-β-CD & HS-β-CD) [35]. Matthij et al. [36] stated the enantiomeric separation of several pharmaceuticals with various acid–basic characteristics (16 basics, 8 acids, and 1 neutral) which were analyzed by employing twin cyclodextrin systems, one of which is strongly sulfated (α, β, γ-HSCD) and one neutral cyclodextrin, i.e., either Dimethyl-β-cyclodextrin (DM-β-CD), TM-β-CD, or Hydroxypropyl-β-cyclodextrin (HP-β-CD), which are examined with these pharmacological substances. In highly sulfated cyclodextrin-based dual-selector systems, the effect of the neutral CD type and concentration on separation is examined. When compared to using just a highly sulfated CD, the CD combinations produce a greater separation for 11 of the 16 basic chemicals. Mixtures with TM-β-CD give better results than those with DM-β-CD and HP-β-CD. The findings indicated that dual CD systems are helpful for the chiral separations of substances that are not properly separated with HS-β-CDs alone. 

A study was undertaken and achieved the development of a high-throughput chiral analysis technique that relies on the simultaneous enantioseparation of nine profens in the normal polarity mode and the reversed polarity mode. The single CD system was used to conduct the conventional polarity mode using TM-β-CD for the enantioseparation of profens in the charged form. In order to operate a dual CD system in the reversed polarity mode, a combination of neutral TM-β-CD and weakly anionic carboxymethyl-β-cyclodextrin (CM-β-CD) was dissolved in the background acidity electrolyte (BEG) modified with hexadimethrine bromide, a polycationic polymer, for the enantioseparation of profens in the uncharged form. They discovered that a dual CD system offers these enantiomers more resolution (R_s_: 1.5–8.0) than a single CD system (R_s_: 1.0–4.4) [37]. Francois et al. [38] carried out the enantiomeric separation of (NASIDs) 2-arylpropionic acids as model compounds carprofen, suprofen, FNP, KTP, NPX, and IBP using two chiral ionic liquids (chiral ILs) (ethyl- and phenylcholine of bis(trifluoromethylsulfonyl)imide) by CE. As a result of these model analytes, the results indicated that these chiral ILs lacked direct enantioselectivity. But most of the time, adding one of the chiral IL to the traditional chiral selectors TMβD or DMβgD resulted in an increase in the resolution. 

Lambert et al. [39] studied the resolution of FL enantiomers by CE with various cyclodextrins (β-CD, HP-β-CD, DM-β-CD, and TM-β-CD). No resolution was obtained with β-CD, HP-β-CD, and DM-β-CD. Only TM-β-CD led to poor separation (R_s_ = 1.32 with 30 mM TM-β-CD in 87.5 mM acetate buffer pH = 5.0). Quek et al. [40] investigated the potential of the CE with capacitively coupled contactless conductivity detection (CE-C_4_D) in the analysis of 13 pharmaceutical products such as IBP and NPX and developed the methods for the separation and quantification of these chemicals in water sources. Satisfactory separation was achieved in less than 14 min. 

Wang et al. [18] simultaneously separated the enantiomers of five profen drugs by micellar electrokinetic chromatography with the combined use of TM-β-CD and chiral cationic ionic liquid, N-undecenoxy-carbonyl-L-leucinol bromide, which formed micelles in aqueous buffers. The enantioseparation of these profen drugs was optimized by varying the chain length and concentration of the ionic liquids’ surfactant using a standard recipe containing 35 mM TM-β-CD and 5 mM sodium acetate at pH = 5.0. This method allowed for the simultaneous resolution of the profen enantiomers with an analysis time of ~45 min and the resolution values were in the range of 2.0–2.4. 

The enantioseparation of some profens, namely FNP, KTP, IBP, and FL in their uncharged form, was investigated using charged CD derivatives in both single and dual CD systems. The use of a cationic single isomer, Permethyl-6-monoamino-6-monodeoxy-β-CD (PMMA-β-CD) (R_s_: 1.64–2) or HS-β-CD (R_s_: 0.6–14), provided a low resolution of some of these profens. However, combinations of these charged CD derivatives can offer excellent possibilities for an enhancement in the selectivity, resolution (4.4–12.5), and peak efficiency for these enantiomers [41]. 

The family of single-isomer, fully sulfated α-CDs, the Hexakis(2,3-diacetyl-6-*O*-sulfo)-α-cyclodextrin (HxDAS), Hexakis(6-Osulfo)-α-CD (HxS), and Hexakis(2,3-di-*O*-methyl-6-*O*-sulfo)-α-cyclodextrin (HxDMS), was synthesized [42,43,44] and used for the initial capillary electrophoretic separation of the enantiomers of nonionic, weak acid, weak base, and ampholytic analytes. The isomeric purity of these chiral selectors was greater than 97%. They were successfully used to separate a variety of the enantiomers of these compounds. 

Octakis(2,3-di-*O*-methyl-6-Osulfo)-γ-CD and Octakis(6-*O*-sulfo)-γ-cyclodextrin, respectively, were synthesized [19,45]. These chiral selectors were used for the CE separation of the enantiomers of nonionic, weak acid such as (IBP, FNP, KTP, NPX, and FL), and weak base analytes. They observed rapid, efficient enantiomer separations for a large number of structurally diverse analytes in acidic aqueous background electrolytes. The enantiomeric separation of six different racemates of the profen family (FL, IBP, INDP, KTP, THP, and SUP) as analytes was carried out in order to test the chiral-selector properties of three members of a new class of cyclodextrin derivatives, Hemispherodextrins (HMs) in CE [15]. The results obtained confirmed the excellent chiral-selector properties of the HMs. Lin et al., synthesized a highly water-soluble new cyclodextrin (CD) derivative 2AHP-β-CD [46]. They explored the capability of the 2-*O*-acetonyl-2-Ohydroxypropyl-β-CD (2-AHP-β-CD) as chiral selectors for the CE resolution of several acidic analytes (IBP, KTP, FL, and NPX) in comparison with the neutral CDs such as DM-β-CD and HP-β-CD. They found that when β-CD and HP-β-CD were added to a BGE containing variable concentrations of CD at pH 5.0, none of the six compounds were separated. When DM-β-CD was added to the BGE, only FL (R_s_ = 0.38) and IBP (R_s_ = 0.86) were partly separated at 40 mM DM-β-CD. When 2-AHP-β-CD was used at 0.49, 0.98, 1.48, and 1.97 g/100 mL buffer, all the compounds were separated. 

Also, Lin et al., synthesized a charged highly water-soluble CD derivative, 6HPTMA-β-CD, and successfully used it as a chiral selector for the enantiomeric separation of some acidic compounds via CZE in an uncoated capillary. The behavior of 6-HPTMA-β-CD was compared with that of the commercially available Quaternary ammonium-β-CD (QA-β-CD) under the same separating conditions. They concluded that 6-*O*-(2-hydroxy-3-trimethylamm-oniopropyl)-β-CD (6-HPTMA-β-CD) was a more effective chiral selector (R_s_: 1.43–2.20) for the enantiomeric separation of these drugs than QA-β-CD (R_s_: 0.0) [47]. 

The usefulness of 6-monodeoxy-6-mono(2hydroxy)ethylamino-β-cyclodextrin (EA-β-CD), 6-monodeoxy-6-mono(2-hydroxy)propylamino-β-cyclodextrin (IPA-β-CD), and 6-monodeoxy-6-mono(2-hydroxy) propylamino-β-cyclodextrin (PA-β-CD) to separate the enantiomers of nonsteroidal anti-inflammatory drugs in nonaqueous CE was demonstrated. Resolution values were always higher with (PA-β-CD) (R_s_: 0.7–9.1) compared to those obtained with (IPA-β-CD) (R_s_: <0.7–7.7) or EA-β-CD (Rs: 2.2–7.3) [48]. 

The possibility of using nonaqueous capillary electrophoresis (NACE) for the enantiomeric purity determination of R-flurbiprofen using 6-monodeoxy-6-mono(2-hydroxy)propylamino-β-cyclodextrin with high resolution (R_s_: 4.8) was demonstrated. A NACE test for FL enantiomer separation and identification in plasma samples using IPA-β-CD and PA-β-CD was also created. These techniques performed well in terms of selectivity, reliability, and precision, and they were well suited for testing enantiomeric impurities in this enantiomer. With an analytical duration of less than 20 min, the enantiomeric separation was quite good (R_s_ = 4.8) [33,49]. 

For the enantioselective analysis of IBP in plasma using HSβD, a direct CE approach was established. Human plasma samples were analyzed for IBP enatiomers in 10 min utilizing the reversed polarity electrokinetic chromatography (CD-EKC) mode [7]. A technique for the simultaneous enantiomeric separation of neutral methyl naproxen (MNPX) and weakly acidic NPX employing neutral cyclodextrin (neutral HP-β-CD) and charged sulfated-β-cyclodextrin (SO_3_-β-CD) as chiral additives via CE was established. In an actual sample under ideal circumstances, they selected 3.5% SO_3_β-CD, 2.0% HP-β-CD, and 10 mM Tris-H_3_PO_4_ (pH 5.5) to determine the chiral separation and purity of NPX. The predicted limit of detection for this approach was 0.7 ppm, and detector linearity was demonstrated over the range of 1.50–100 ppm of the different enantiomers of NPX [50]. The Application of two new types of cyclam-capped-β-CD-bonded silica nanoparticles, Mono-(8-benzenesulfonamidoquinoline-2-ylmethyl)-substituted cyclam (MCCD-HPS) and 1,8-di-(2-hydroxymethylpridine-6-ylmethyl)-substituted cyclam-capped(3-(2-*O*-â-cyclodextrin)-2-hydroxypropoxy)-propylsilylp-pendedsilica (DCCD-HPS), as chiral stationary phases (CSPs) in capillary electrochromateography (CEC) for the separation of many chiral chemicals, including KTP and INP, has been described [51]. They have good enantiomeric selectivities, and INP obtained baseline enantioseparations for numerous solutes (R_s_: 5.33).

Liu et al. [8] developed a straightforward procedure for the chiral separation of ephedrine and associated chemicals using DM-β-CD-modified CE. Ephedrine and its related compounds’ enantioseparation was accomplished under ideal circumstances. Additionally, this technology has been effectively used to identify these chemicals in a number of medications, including compound PSEHHCl tablets and compound fritillaries extract tablets. For the detection of ephedrine and pseudoephedrine enantiomers in dietary supplements and related products, three complementary CE techniques have been established [52]. How to use the CE technique to identify the main stereoisomers of the ephedrine alkaloid in a range of samples that contain ephedra has been established. Sensitivity for these analytes was significantly increased by a high-sensitivity UV detection cell. The application of complementary CE techniques increases peak identity certainty, and it has been predicted that this strategy can be used to solve other analytical problems.

Patel et al. [53] successfully employed Heptakis(6-amino-6-deoxy)-β-cyclod-extrin (per-6-NH_2_-β-CD) as a chiral selector for the enantioseparation of several anionic analytes, including IBP, FNP, and KTP via CE, (R_s_: 1.6, 4.3, 4.0), respectively. Their findings support the hypothesis that this chiral selector’s enantiorecognition mechanism relies primarily on hydrogen bonding and sterically regulated guest inclusion in the per-6-NH_2_-CD cavity. In comparison to KTP and FNP, it appeared that IBP with one phenyl ring had provided a superior fit to the cavity of this chiral selector [54]. Pharmaceutics’ IBP and FL were measured using CE and spectrophotometric detection [55]. The procedure has been verified, and the outcomes were precise and correct. Using this enhanced background electrolyte, the analysis was completed in less than 5 min. Table 1 shows the abbreviation of the chiral selectors and their description, while Table 2 provides a review of the literature on the use of CE for chiral drug separations.

### 1.3. HPLC for Chiral Drugs Analysis

The most widely used method for chiral analysis is HPLC, which is also the most flexible, effective, and significant instrument in a variety of domains, including pharmaceutical quality control and drug pharmacokinetic research.

This is due to its benefits over alternative separation processes, such as its resilience, high precision, wide range of detectors, diversity of separation modes, abundance of stationary phases, etc. [62]. In general, there are three types of chiral separation techniques used in HPLC: direct separation using a chiral stationary phase, like polysaccharides or antibiotics. Using chiral mobile-phase additives (CMPAs) in the mobile phase to form adducts with the enantiomeric analytes directly (method) and separating the diastereomers produced by precolumn derivatization with chiral derivatization reagents (CDR) like 4-(*N*,*N*-dimethylaminosulphonyl)-7-(3-aminopyrrolidin-1-yl)-1,3-NBD-APY, 4-(aminosulphonyl)-7-(3-aminopyrrolidin-1-yl)-2,1,3-benzoxadiazole (ABD-PY), and 7-(3-aminopyrrolidin-1-yl)-2,1,3-benzoxadiazole (indirect method).

The physical and chemical features of the pair of diastereomers produced by the indirect technique can be distinguished chromatographically on a typical HPLC column. With regard to selectivity, sensitivity, and flexibility, this methodology uses an appropriate CDR that contains an appropriate chromophore or fluorophore as a method for enantiomer trace determination, particularly in biological specimens. The diastereomeric approach offers two advantages over the direct method while requiring more manipulation and time in general: (i) in the presence of numerous other analytes, the detection of a trace analyte can be accomplished by observing the elution order of the diastereomers produced by a CDR, choosing the chiral reagent enantiomer, or altering the components of the mobile phase; and (ii) the commercial availability of a large variety of CDRs for derivatizing various functional groups, along with the use of optically pure isomers, whose corresponding racemates occasionally serve as enantiomeric analytes or as CDRs due to the reciprocity of two reactants in a derivatization reaction, all contribute to the versatility of the indirect approach. For instance, a chiral carboxylic acid reagent can derivatize amine racemates when employed as a CDR. Alternatively, a matching chiral amine reagent can be used as a CDR to enantioseparate amine racemates.

The chiral center must be close to the reactive functional group, chirality must lead to efficient resolution, a strong chromophore or fluorophore must react to a sensitive detection method such as fluorescence or chemiluminescence, and the diastereomeric derivatives must have great stability. The enantioselectivity, stability, sensitivity, and reactivity (reaction rates and derivatization conditions) of the reagent are among a CDR’s analytical qualities along with its diastereomers. Reversed-column techniques may be more appropriate for the study of biological samples; however, it should be kept in mind that the enantioselectivity of the CDR utilized is always tied to a chosen chromatographic column system, whether a normal-phase column or reversed-phase column. The enantioselectivity of a particular CDR may also be somewhat influenced by other variables, including chromatographic conditions (such as the mobile phase, temperature, pH, type of buffer, except for the stationary phase) and structural differences between enantiomeric analytes [66,67].

The choice of an appropriate CDR will have a significant impact on a number of structural parameters, including the distance between the chiral centers, bulk dissymmetry, and polarity of substituent groups around the chiral center, which all contribute to the successful resolution of the diastereomers. The degree of relative diastereomer chromatographic resolvability, or (R_s_ value), is influenced by the conformational and configurational characteristics of the molecules as well as other factors [62].

Due to its high sensitivity, selectivity, and accessibility of a variety of commercial columns, HPLC with a CSP is the most effective direct method of chiral analyte separation. Undoubtedly, a crucial component of chiral HPLC is the design and development of a CSP capable of efficiently chiral recognizing a variety of enantiomers [68,69]. The success or failure of a chromatographic separation in HPLC is determined on the best CSP selection. Currently, a variety of CSP types, including macrocyclic antibiotics and CSPs derived from cellulose and amylose, have demonstrated their utility for chiral separation.

These CSPs have been used to separate a variety of racemic chemicals, including aromatic alcohols, amides, pyriproxyfen, amino alcohols, diols, -blockers, carboxylic acids, and other unrelated compounds [46]. When employed as eluents in the mobile phase, derivatized cellulose- and amylose-based CSPs are not well matched to all solvents. The polysaccharide derivatives themselves cannot be used as eluents in some solvents classified as forbidden HPLC solvents, such as ethyl acetate, tetrahydrofuran, methyltert-butyl ether, dichloromethane, and chloroform. Therefore, unless the harmful solvent is removed and the analyte itself is dissolved in conventional mobile-phase solvents, a reaction conducted in any of the forbidden HPLC solvents cannot be directly or online monitored by HPLC. The polysaccharide derivatives have been immobilized on a silica matrix and are frequently utilized as the chiral stationary phase to address this flaw. This method of immobilizing the polymeric chiral selectors on the silica support is thought to be effective in granting the CSP a broad range of solvent compatibility, increasing the number of solvents that can be utilized as [70,71].

A CSP with a lengthy and interesting history is CHIRAL-AGP. Hermansson first presented it in 1983 for the direct resolution of racemic pharmaceuticals by HPLC under RP circumstances [72]. Since then, this CSP has been extensively used in a variety of application fields, including the identification of active chiral chemicals and their metabolites in biological media, the investigation of bioactive chiral compounds in plants, and pharmacokinetic studies of chiral pharmaceuticals. Numerous studies have focused on CHIRAL-AGP’s ability to resolve enantiomers of different categories, and as a result, several verified pharmacological analysis methodologies have emerged [57,73].

More recently, it was demonstrated that the bound phases of macrocyclic antibiotics were effective in generating enantioseparations. One of the earliest bonded CSP (Chirobiotic V^TM^) macrocyclic glycopeptide antibiotics to be developed was vancomycin. In comparison to the protein-based CSP, the macrocyclic antibiotic-bound phases exhibit higher capabilities and are more stable. While a CSP offers excellent enantiomer separation, using direct techniques to resolve some enantiomeric compounds like amines and carboxylic acids is frequently challenging. Other drawbacks of the CSP column are that it is more expensive, has a shorter lifespan, and it is challenging to choose the best column [48,62].

#### HPLC for Chiral Drug Separations

Liquid chromatography combined with tandem mass spectrometry (LC-MS-MS) was used [63] to analyze IBP in human plasma. For the resolution of IBP, the CHIRALPAK AD-RH column was utilized. This method’s great selectivity as a result of using the MS detection technology is its key advantage. This type of detection virtually eliminates interference from endogenous substances and other drugs that are provided concurrently, making technique development simple. Ghanem and Aboul-Enein compared Chiralpak IA and Chiralpak AD, finding that Chiralpak IA had a weaker chiral detection capacity and a worse ability to resolve chiral patterns. Moreover, the Chiralpak IA is more flexible than the Chiralpak AD in terms of monitoring reactions carried out in prohibited HPLC solvents like DCM; Chiralpak AD: (R_s_ = 1.6–6.68), Chiralpak IA: (R_s_ = 1.9–3.1).

Ahmed et al., created and evaluated native 2-HP-β-CD and β-CD chiral stationary phases for HPLC [74]. A wide variety of optically active substances, including IBP, WAF, and NPX, were well received by the two columns, which exhibited good enantioselectivity. The native β-CD phase allows for the baseline separation of four pairs of enantiomers, while the 2-HP-β-CD phase allowed for the identification of both enantiomers’ existence and incomplete separation for eight compounds.

According to Michishita et al., CHIRAL-AGP is unquestionably a very potent chiral column that offers an exceptional success rate for enantiomers of various kinds [6]. However, because of its poor sample-loading capacity, its use is restricted to the analytical level. On the other hand, Fanali et al., discovered that CHI-TBB is a suitable chiral stationary phase for the enantioseparation of a number of acidic chemicals. They were able to acquire good enantioresolutions for acidic chemicals with medicinal and environmental relevance by using CHI-TBB in a nano-LC system, sometimes with quick analysis times, high peak efficiency, and very little consumption of both organic solvents and chiral stationary phase [59,65].

Patel et al., analyzed the enantiomers of FL, FL-OH, and FL-MOH utilizing four different methods for chromatographic separation and resolution: reversed-phase HPLC following (*R*)-l-(naphthen-lyl) ethylamine precolumn derivatization; utilizing hydroxypropyl-13-cyclodextrin as a chiral mobile-phase additive in reversed-phase HPLC; employing either ChiraI-AGP or Chiralpak AD for chiral-phase HPLC. Out of all the methods, only the direct method using the Chiralpak AD CSP was able to separate and resolve the enantiomers of all three analytes in under 45 min. For FL, FL-OH, and FLMOH, respectively, enantiomer resolution values of 1.67, 3.67, and 3.44 were achieved [53].

An effective substitute for human serum albumin (HAS) columns containing silica particles or a Glycidyl methacrylate/ethylene dimethacrylate monolith for chiral resolution was found to be a silica monolith containing immobilized human serum albumin. Comparing the HSA silica monolith to HSA columns that contained silica particles or a GMA/EDMA monolith, the HSA silica monolith provided higher retention and higher or equivalent resolution and efficiency. In applications needing high flow rates, where the silica monolith can provide adequate efficiencies and tolerable backpressures, this sort of column should be especially helpful [54].

In the chiral evaluation of a collection of acidic medicines employing several nonstandard or forbidden solvents in a reaction mixture, Ghanem et al. [55] established the solvent versatility of the Chiralpak IA and IB on silica. The findings showed that the range of solvents that can be applied to these CSPs has undoubtedly been expanded, and that novel enantioselectivity may emerge when using unconventional or illegal solvents. Enantioselective separations are challenging to transfer from the older, traditionally coated CSPs (Chiralpak AD-H and Chiralcel OD-H) to the newer, immobilized versions due to differences between the immobilized and coated CSPs (Chiralpak IA and IB). Aboul-Enein successfully resolved some enantiomeric profen mixtures, including KTP, FL, Pirprofen (PIP), and Tiaprofenic Acids (TIPA), on the tartardiamide-DMB chiral stationary phase [75]. For the baseline isolation of these profens, hydrogen bonding between the analyses and the CSP was crucial.

An immobilized chiral stationary phase called Chiralpak IB, a cellulose derivative, was created by Ghanem et al. [76]. This immobilized version of Chiralcel OD was used and proved to be adaptable for the monitoring of the lipase-catalyzed kinetic resolution of racemates in unconventional organic solvents. Chiral CEC and nano-HPLC separations can be performed using monolithic columns with physically and chemically linked β-cyclodextrin derivatives [77]. Within three months of testing, prepared monolithic columns demonstrated the ability to separate the model mixes of significant drug enantiomers and proved to be adequately stable.

Ye et al. [78] accomplished the enantioresolutions of eight NSAIDs by RPHPLC with HP-β-CD as a CMPA. Because HP-β-CD is suitable for practically all 2-Arylpropionic acid derivative (2-APA) NSAID enantiomers and has strong solubility in both water and organic solvents, optimizing the chromatographic conditions is simple. By just adjusting the buffer-to-methanol ratio in the mobile phase, this approach can separate the majority of 2-APA NSAID enantiomers under chromatographic conditions, is adaptable, straightforward, and favorable economically. The technique was successfully used in an enantioselective skin-permeation investigation to determine the racemic 2-APA NSAIDs. In addition to being produced, CalixBz-Cl was structurally analyzed and employed as an HPLC selector. Different medications, including IBP, were utilized in selectivity trials during that phase. The chromatographic results showed that silica-bonded calixarenes have properties of the reversed phase [79]. The HPLC method established by Péhourcq et al., has been successfully used to measure FL enantiomers enantioselectively in rat plasma on a bonded vancomycin chiral stationary phase (ChirobioticV^TM^). It was consistent, accurate, and stereotypical [78]. It explored chiral stationary phases by comparing Kromasil^®^ CHI-II and Chirex^®^ 3005. Due to excellent resolution and low backpressure as compared to other columns, they chose the Kromasil CHI-II column as a stationary phase to separate the S-ketoprofen enantiomer from the racemate mixture.

Vermeulen confirmed a stereospecific HPLC technique based on acid glycoprotein-bound chiral stationary phase to describe the pharmacokinetic characteristics of each enantiomer of IBP [66,66]. The approach was exact, particular, accurate, and repeatable. The limits of quantification for *R*-(2)- and *S*-(1)-IBP were 1.16 and 1.37 mg mL^−1^, respectively. A novel 2,3-methylated-3-monoacetylated 6-*O*-tertbutyldimethylsilylated -CD derivative was created by Bayer et al., and chemically attached to monolithic silica HPLC columns that had undergone aminopropyl derivatization [80,81]. Out of 32 compounds, 14 could be separated into their enantiomers.

Bosáková et al., used the chiral selector vancomycin (Chirobiotic V and Chirobiotic V2) for the enantioseparation of profens. The higher retention of profens on the Chirobiotic V2 column was not always accompanied by an improvement in their chiral separation in the RP mode [82].

Sakaguchi et al., devised fluorous derivatization followed by fluorous-phase LC separation, which makes use of the affinity between perfluoroalkyl compounds for the highly selective and quantitative isolation of diverse analytes [83]. They demonstrated how this method may be used to clinically determine the presence of naproxen in human plasma. In order to very selectively retain just the derivatized compounds in the fluorous LC column, samples were precolumn-derivatized with a nonfluorescent fluorous amine. Thus, at the proper retention durations, only the retained fluorescent and fluoro-labeled analytes were fluorometrically identified. These two substances’ detection thresholds were lower than 11 × 10^−15^ mol on the column. Guo et al. [84] introduced a method for the chiral separation of KTP enantiomers on an achiral C8 column using norvancomycin as a chiral selector added to the mobile phase. Their research has demonstrated the effectiveness and simplicity of the optimized CMPA approach for the chiral separation and quantification of (*R*) and (*S*)-KTP.

On an achiral ODS column, *R*-, *S*-naproxen was chirally separated using methyl-β-CD as a mobile-phase additive. The technique was successfully adapted to a nano-LC system, which decreased the need for pricey CMPAs. Baseline NPX enantiomer separation was achieved in nano-LC (α = 1.13, R_s_ = 1.69) [85]. As a brand-new chiral fluorescence derivatization agent for the investigation of carboxylic acid enantiomers like KTP and IBU, DNS-Apy was created. Low detection limits and excellent carboxylic acid enantiomer resolution were achieved (pico mol) [86]. The techniques for chiral drug separations using HPLC are listed in Table 3.

### 1.4. Nonsteroidal Anti-Inflammatory Drug (NSAID)

One of the most popular and significant classes of analgesic anti-inflammatory medications is that of 2-arylpropionic acid NSAIDs. These medications can be identified by the presence of a chiral carbon atom close to the carboxylic acid group. It is well known that different 2-APA enantiomers have various therapeutic effects. Diverse chiral medicines have been subjected to a number of direct/indirect liquid chromatographic techniques for their potential resolution and enantiomeric analysis [58].

#### 1.4.1. Ibuprofen

**Figure 2:**
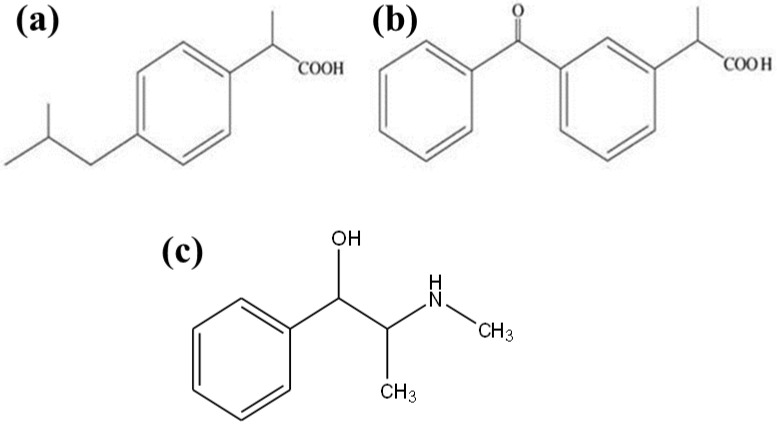
Chemical structures of (**a**) KTP, (**b**) IBP, (**c**) PSEH; several steps of the analytical procedure can be integrated and automated within the system.

[(±)-(*R*,*S*)-2-(4-isobutylphenyl) propionic acid]/IBP is a chiral NSAID medicine commonly used to treat a number of rheumatic and musculoskeletal illnesses. It is mostly used to treat arthritis, primary dysmenorrhea, fever, and as an analgesic, particularly when there is an inflammatory component [21]. Ibuprofen is a racemic combination, although the (+)-(*S*)-enantiomer is primarily responsible for its anti-inflammatory effects. IBP is extensively metabolized by the glucuronidation and oxidation pathways, with a preference for (*S*)-IBP. Due to the considerable differences between the plasma concentrations of (*R*)- and (*S*)-IBP, stereoselective pharmacokinetic characteristics are produced [33]. Figure 2a depicts IBP’s chemical structure.

#### 1.4.2. Ketoprofen

A chiral NSAID with a pKa of 4.55, known as KTP, (*R*,*S*)-2-(benzoylphenyl) propionic acid, has analgesic and antipyretic properties. It decreases the formation of prostaglandins in the tissue while inhibiting the activity of cyclooxygenase (COX_2_ and COX_1_). Despite the fact that its (+)-*S*-enantiomer is the only one with therapeutic efficacy, the racemic combination is nevertheless sold [31]. It is interesting to note that in the presence of some enzymes and microbial enantiomer–enzyme complexes with endogenous triacylglycerols, the inactive R enantiomer can change configuration to become the active enantiomer. Therefore, administered profen accumulates to some amount in the body, raising the possibility of hazardous side effects. Therefore, quick, focused, and sensitive techniques are needed for the pharmaceutical sector to monitor the (*S*)-KTP’s enantiomeric purity. Typically, chiral HPLC is used to achieve this [89]. Figure 2b depicts KTP’s chemical structure.

### 1.5. Pseudoephedrine Hydrochloride

{[*S*/*R*-(R*,R*)]-a-[1-(methylamino)ethyl]-benzenmethanol}/PSEH-HCl is an adrenergic drug. It is a commonly used nasal and bronchial decongestant for the therapeutic treatment of the common cold, sinusitis, hay fever, bronchitis, and respiratory allergies, either alone or in conjunction with other medications [90]. Both a direct- and indirect-acting sympathomimetic amine, PSEH is categorized. PSEH has been detected in biological samples and pharmaceutical preparations using a variety of techniques, including HPLC, GC, and CE [91]. Figure 2c depicts PSEH’s chemical structure.

### 1.6. Microfluidics System

Analytical system miniaturization has been a long-running research topic thanks to advancements in microfabrication techniques [92]. As reagent amounts decrease from milliliter to microliter and nanoliter levels as a result of this progress, analysis costs will generally decrease. Additionally, in terms of response time, analytical throughput, automation, and mobility, downsized systems are linked to increased efficiency [93]. One system can accommodate several assays without increasing the device’s size or complexity.

Micro total analysis systems (μTAS), sometimes known as “Labs on a Chip,” were developed as a result of these concepts. The functions of large analytical equipment can be carried out by this system in compact components. To put it another way, µ-TAS is the downsizing of an entire analytical process from sample preparation through reaction and separation to detection [94]. In the majority of the technologies currently in use, separation plays a crucial role generally and is anticipated to be the primary step in tiny systems. The separation phase must be combined with techniques that come before or after it, such as derivatization, enrichment, and sample cleanup methods.

The ability to handle fluidics on the nanoliter and even picoliter scale, which has expanded the scope of microTAS and given it the name microfluidics, is the other significant characteristic. Consequently, the term “microfluidics” refers to the science and technology of controlling tiny volumes of fluid, ranging in size from microliters to femtoliters, through networks of channels with dimensions of 5–500 µm. The tiny analytical system had to overcome numerous obstacles, including those related to automation, sampling, sample preparation, sample introduction, and detection. In a high-throughput application where samples fluctuate, such as continuous online process monitoring, the interface of chips to macroscale systems is also seen as a big difficulty [95]. Because of the decrease in linear dimensions, the chemical makeup of the surface and surface preparation techniques are significant. The surface to volume ratio in a capillary is higher than in a regular tube or a column, and it rises in a microreactor (10,000 to 50,000 m^2^m^−3^, compared to 1000 m^2^m^−3^ in a conventional laboratory vessel) [96,97].

Typically, a network of micron-sized channels (10–500 µm in diameter) carved into a planar surface are used in microreactors to manage small amounts of chemicals. Reactors have been invented recently, and several of them are currently readily available on the market. How applicable it is depends on the reactor’s size, the chemical and physical properties of the material used to construct it, the kind of chemicals and solvents added to the system, and its size [98].

**Figure 3:**
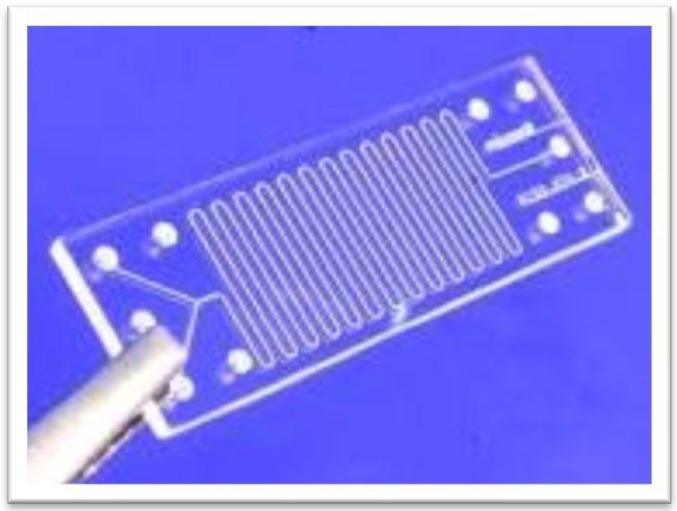
A microreactor produced by the powder-blasting method. The chemist’s point of view is to drive solutions through the reactor by hydrodynamic pumping.

Microreactors have been built from a variety of materials, including glass, silicon, stainless steel, metals, and polymers. Chemical compatibility, ease of manufacture, and reproducibility are crucial factors to take into account when selecting a material. Being chemically inert, allowing electroosmotic flow (EOF) with many common solvents, allowing the use of a variety of visible-light-sensing technologies, and having well-established production procedures make glass a popular material choice. Various fabrication techniques, including photolithography, hot embossing, powder blasting, injection molding, and laser microforming, where the channels are etched into the material using strong lasers, are available depending on the material utilized [98]. Figure 3 depicts an illustration of a microreactor created by powder blasting. Microreactors’ channels can be used to pump fluids through employing techniques like capillary flow, electrokinetic pumping, and hydrodynamic pumping. The simplest method for operating a microreactor is from the synthesized.

By employing EOF, in which a voltage is given to the reagent and collection reservoirs, fluids can be moved in glass microreactors. Since there are no moving parts involved in this type of “pumping”, it offers some advantages over hydrodynamic pumping, including the ability to be easily downsized and meticulously controlled by a computer program. But EOF (needs polar solvents, depending on the solute concentration) may only be used in analytical size reactors and may result in undesirable electrochemical transformations and/or separations. Techniques for capillary flow have a limited application in chemical synthesis [99]. Although fluid levels may be precisely controlled, the capillary reactors’ transferred volumes are quite small. Regardless of the pumping method, the flow inside a chip-based microreactor’s channel network is typically laminar, and reagent mixing happens by diffusion and convection. Diffusion is typically quite effective due to the small size of the devices, and mixing takes place within milliseconds.

To create desired products with a high yield and selectivity while accurately managing reactor operations, improving the mixing performance in microreactors is another crucial challenge. To reduce the mixing time, the selectivity’s shear rates to reactant fluids are crucial. To partition reactant fluids into numerous small fluid segments using the channel shape of micromixers is the first of two main principles that are commonly used. Reactant fluids have to be separated into numerous laminated fluid segments by the shape of the inlet channels into the mixing chamber in order for a mixing method based on this mixing principle to work. By reducing the number of channels, this mixing shortens the length of diffusion, which is necessary for rapid mixing. But the reduced channel also causes a high-pressure drop in the channel, which limits the flow rate and reduces the productivity and operability [100].

The second main principle entails breaking up reactant fluids into smaller fluid segments, which speeds up the reactant fluid collision and reduces the mixing time. The simplest application of this blending principle is the collision of two fluid streams. The examination of the relationship between design elements such as channel diameters and flow rates in the mixers, flow pattern, and mixing performance in the micromixers has used T- and Y-shape microchannels as examples of micromixers that use this mixing concept [100]. Table 4 displays a few of the microreactor-performed reactions. The flow folding kind of microreactor was employed in this study to improve mixing.

### 1.7. Monolithic Column

The column, which is the primary part of an HPLC system, enables the division of a complicated mixture into separate species based on the selectivity and column performance. For the separation of chemicals in many sectors, particle-packed HPLC columns have been used. An amount of 3–5 µm porous silica microparticles makes up the majority of the filling in these columns. The size, dispersion, and quality of the packing of the particles inside the column have a significant impact on the extraction efficiency of the column [118]. Most standard particulate-based HPLC columns take a long time to analyze (usually 5 to 30 min), which is necessary for a full separation cycle, because the high backpressure prevents these columns from operating at high flow rates (>2 mL min^−1^).

Very small particles (less than 2 µm in diameter) packed onto a column can considerably improve the separation efficiency and cut down on the analysis time. But, compared to typical HPLC systems packed with 3–5 µm particles, the usage of small particle columns requires higher pressure to overcome the pressure restrictions. Monolithic columns, an alternative HPLC technology to the traditional chromatographic methods, have recently been introduced. A single piece of porous crosslinked polymer or porous silica makes up monolithic columns. These columns can be created in a variety of ways, including as porous rods, thin capillaries, thin membranes, or thin discs. In order to accomplish high-speed separations, low column backpressure, and quick mass-transfer kinetics while maintaining good separation, monolithic columns are frequently used in HPLC. A chromolith column is unique in that its monoliths have a definite bimodal pore structure, with macropores measuring 2 µm in diameter and mesopores measuring 13 nm on average. The monolithic column’s overall porosity is in the 80 percent to higher range, with the greater fraction accounting for the macropores. The mesopores can produce a huge, focused surface area that can be as high as 300 m^2^g^−1^. Due to the high porosity, there are many theoretical plates per unit pressure drop and high permeability. With monolithic silica columns compared to a particle-packed column, a lower plate height and lower pressure drop are produced due to a high permeability and small diffusion path length provided by the presence of large through-pores and relatively small skeletons, allowing for faster separations with current instrumentation [31,119,120].

In reversed-phase HPLC under isocratic conditions, the column efficiency of a 5 µm C18-bonded silica column and a monolithic C18-bonded silica column was examined. A plate height H of 10–15 µm was observed in the particle-packed column at a linear velocity of 1 mm s^−1^. The monolithic column’s Van Deemter plot took the same path but almost paralleled the abscissa up to a linear velocity of 7 mms^−1^. Due to the high column backpressure at this high velocity, the packed column could not be operated. The column backpressure for the monolithic RP column was three to five times lower than for the particle-packed column [121,122].

High-throughput drug and metabolite analysis has been the main focus of monolithic column applications in the pharmaceutical industry. High flow rates and monolithic columns measuring 50 mm by 4.6 mm were frequently used to achieve the high-speed separations. The total proficiencies for such a column were comparable to or a little higher than those for a 50 mm × 4.6 mm, 3 μm C18 column, but still much inferior than a conservative 5 μm, 250 mm × 4.6 mm or 3 μm, 150 mm × 4.6 mm C18 column, which are most commonly used in procedure progress. Traditional silica “Particle-Based” columns and columns are contrasted. The micrograph depicts the monolithic silica column’s silica skeleton, which is composed of a network of 2 µm pores. A fine network of 13 nm pores, which are too small to be visible in the micrograph, may be seen inside the skeleton [123,124].

## 2. Conclusions

In conclusion, chiral separation techniques are vital in many scientific and industrial fields, with major consequences for chemistry, pharmacy, biochemistry, and medicine. Many applications require enantiomer separation and analysis. Chiral separation procedures ensure safe and successful medication development. Chiral enantiomers can have different biological activity and medicinal effects. Thus, reliable enantiomeric purity determination is essential for medication design, pharmacokinetics, pharmacodynamics, and side effects. Chromatography, electrophoresis, and crystallization isolate and characterize enantiomers for biological activity assessment. Enantiomeric quantification is essential for pharmaceutical formulation quality and uniformity. Chiral separation also helps monitor and remove chiral contaminants in environmental study. Chiral pollutants with different toxicities and degradation rates must be assessed and removed individually. Industrial chiral intermediate and fine chemical synthesis benefit from chiral separation. Separating enantiomers facilitates the manufacture of stereochemically specific, high-value compounds, improving chemical process efficiency and selectivity. Chiral separation allows for the manufacture of enantiomerically pure chemicals, which can improve industrial operations economically and practically. Capillary electrophoresis (CE) and high-performance liquid chromatography (HPLC) are powerful techniques for the analysis of chiral drugs. They investigate chiral drug metabolism, receptor interaction, and stereoselective toxicity. They help evaluate chiral drug pharmacokinetics, pharmacodynamics, and stability during drug development. Microfluidics technologies and monolithic columns have expanded chiral separation possibilities. Miniaturization, high throughput, and precise fluid flow control allow microfluidics systems to efficiently and quickly resolve enantiomers.

In summary, chiral separation techniques are crucial in many scientific and industrial fields. Identifying and isolating chiral substances improve treatment outcomes, environmental protection, and industrial operations by revealing their features and effects. Chiral separation technology, such as CE, HPLC, microfluidics, and monolithic columns, continues to improve enantiomeric analysis and drug discovery, quality control, and patient care.

## Figures and Tables

**Table 1:** The abbreviation of the chiral selectors and their description.

Chiral Selector	Description
TM-β-CD	Trimethyl-β-cyclodextrin
DM-β-CD	Dimethyl-β-cyclodextrin
HP-β-CD	2-hydroxypropyl-β-cyclodextrin
PMMA-β-CD	Permethyl-6-monoamino-6-monodeoxy-β-CD
HxDAS	Hexakis(2,3-diacetyl-6-*O*-sulfo)-α-cyclodextrin
HXS	Hexakis(6-*O*-sulfo)-α-CD
HxDMS	Hexakis(2,3-di-*O*-methyl-6-*O*-sulfo)-αcyclodextrin
ODMS	Octakis(2,3-di-*O*-methyl-6-*O*-sulfo)-γ-CD
OS	Octakis(6-*O*-sulfo)-γ-cyclodextrin
HMs	Hemispherodextrins
QA-β-CD	Quaternary ammonium-β-CD
2-AHP-β-CD	6-monodeoxy-6-mono(2-hydroxy)propylamino-β-cyclodextrin
6-HPTMA-β-CD	6-*O*-(2-hydroxy-3-trimethylammoniopropyl)-β-CD
PA-β-CD	6-monodeoxy-6-mono(2-hydroxy)pro- pylamino-β-cyclodextrin
EA-β-CD	6-monodeoxy-6-mono(2-hydroxy)ethylamino-β-cyclodextrin
IPA-β-CD	6-monodeoxy-6-mono(2-hydroxy)propylamino-β-cyclodextrin
HS-β-CD	Heptakis-6-sulfato-β-cyclodextrin
SO_3_-β-CD	Sulfated-β-cyclodextrin

**Table 2:** The use of CE for chiral drug separations.

Drug	Matrix	Chiral Selector	Analytical Data: LOQ, (R_s_)	Detector	Ref.
KTP	Standard solutions	TM-β-CD	0.002 mg/L, (3.77)	UV	[37]
KTP	Human serum	TM-β-CD	0.1 mg/L, (4.32)	UV	[47]
IBP, FBP, KTP, NPX	Human serum	TM-β-CD	0.25–1.0 μg/mL, (1.88–3.70)	UV	[34]
IBP	Human serum, urine	TM-β-CD	Serum:(2.19), urine (2.25)	UV	[56]
IBP, IBP-OH, IBP-COOH	Human serum, plasma	TM-β-CD	0.11 mg/L in plasma, 1.0–1.1 mg/L in urine, (1.04–2.26)	UV	[40]
KTP	Human plasma, urine, synovial fluid	TM-β-CD	Plasma:0.25 mg/L (3.87), synovial fluid: 0.50 mg/L (4.29)	diode array	[41]
INDB	Human serum	TM-β-CD	0.2 g/mL, (1.5)	UV	[42]
FNP, FL, IBP, KTP	Standard solutions	HS-β-CD TM-β-CD	HS-β-CD: (1.3–1.4) HS-β-CD: - TM-β-CD: (2.9–6.1)	UV	[5]
IBU	Standard solutions	HS-β-CD, TM-β-CD	HS-β-CD: (0.7), HS-β-CD+ TM-β-CD: (3.3)	UV	[44]
16 basics, 8 acids, 1 neutral	Standard solutions	HPCD, TMCD, DMCD, HSCD	Neutral CD: (0.00–4.05), HSCD: (0.00–19.40)	UV	[32]
IBP, FNP, FL, SUP, INP, KTP, P, IP, TIPA, NPX	Standard solutions	TM-β-CD, CM-β-CD	TM-β-CD: (1.0–4.4), TMβC-M-β-CD: (1.5–8.0)	UV	[44]
CAP, NPX, SUP, KTP	Standard solutions	TM-β-CD DM-β-CD	(0–1.26)	UV	[45]
FL	Standard solutions	β-CD, HPβ-CD DM-β-CD TM-β-CD	DM-β-CD (0.0) TM-β-CD (1.32)	UV	[46]
IBP, NPX	Standard solutions	HP-β-CD TM-β-CD γ-CD	61–1676 mg/L Urine: 1.0 mg/L (4.06)	C4D	[47]
IBP, FNP, INP, S UP, KTP	Standard solutions	L-UCLB- TM-β-CD	L-UCLB-TM-β-CD (2.0–2.4)	diode array	[30]
FNP, KTP, IBPF L	Standard solutions	PMMA-β-CD HS-β-CD	PMMA-β-CD:(1.64–2), PMMA-β-CD HS-β-CD: (4.4–12.5)	UV	[57]
IBP, FNP, KTP, NPX	Standard solutions	HxDAS	(0–3.1)	UV	[49]
CAP, NPX, FNP, KTP, IBU, FLB	Standard solutions	HXS	-	UV	[42]
IBP, FL, FNP, KTP	Standard solutions	HxDMS	(0–1.3)	UV	[58]
IBP, NPX, FNP, K TP	Standard solutions	ODMS	(0.0–3.1)	UV	[30]
IBP, FL, FNP, KT P	Standard solutions	OS	(0.5–1)	UV	[59]
FL, IBP, INP, KTP, SUP, THP	Standard solutions	HMs	-	UV	[35]
IBP, KTP, FL, NPX, WAF, OXN	Standard solutions	DM-β-CD HP-β-CD 2-AHP-β-CD	DM-β-CD & HP-β-CD (0), DM-β-CD FLP (0.38), IBP (0.86), 2-AHP-β-CD: (0.79)	UV	[60]
NPX, IBP, INP, K TP, FL	Standard solutions	6-HPTMA-β-CD QA-β-CD	6-HPTMA-β-CD: 9 Mm (1.43–2.20) QA-β-CD: 9 mM (0.00)	UV	[61]
FNP, FL, IBP, IN P, KTP, SUP, TIP A, WAF	Standard solutions	EA-β-CD, IPA-β-CD PA-β-CD	EA-β-CD: (2.2–7.3) IPA-β-CD: (<0.7–7.7)	UV	[48]
FL	Standard solutions	IPA-β-CD	0.1%,(4.8)	diode array	[62]
FL	Human plasma	PA-β-CD	0.2 mg/mL (4.8), Dual CD, (0.00–40.00)	diode array	[25]
IBP	Human plasma	HS-β-CD	0.2 mg/L	UV	[6]
NPX& MNPX	Standard solutions	HP-β-CD, SO_3_-β-CD	6.8 × 10^−4^ g/L	UV	[63]
KTP, INP	Standard solutions	MCCD-HPS, DCCD-HPS	KTP (1.22) INP (5.33)	diode array	[64]
EPD	Standard solutions	DM-β-CD	70–161 ng/mL	UV	[7]
EPD	Standard solutions	DM-β-CD HP-β-CD S-β-CD	-	UV	[65]
IBP, FNP, KTP	Urine, standard solutions	β-CD	Urine: 18–38 standard solutions: 1.7–10	UV	[54]
IBP, FL	Standard solutions	α-CD	IBP: 0.5 mg/L FL: 0.1 mg/L	UV	[55]

**Table 3:** The use of HPLC for chiral drug separations.

Drug	Chiral/Achiral Reagent	Type of Chiral Selector	Analytical Data: LOQ, (R_s_)	Detector	Ref.
IBP	Chiralpak-RH	CSP	0.12 g/mL	MS	[63]
KTP, IBP, FN P, FL, WAF, NPX, CAP	Chiralpak-IA, Chiralpak-AD	CSP	IA: (1.9–3.1) AD: (1.6–6.68)	UV	[51]
IBP, WAF, NPX	Native β-CD 2-HP-β-CD	CSP	Native β-CD (0–1.3) 2-HP-β-CD (0)	UV	[74]
FL	Chiral-AGP	CSP	(1.50)	UV	[6]
NPX, IBP, CAP, KTP	CHITBB	CSP	(0.54–2.40)	UV	[65]
FL, FL-OH, FL-MOH	Chiralpak-AD	CSP	(1.67, 3.67, 3.44)	UV	[53]
IBP, WAF	HSA	CSP	(1.8–2.4)	UV	[54]
KTP, IBP, FNP, FL, CAP	Chiralpak IA Chiralpak IB	CSP	Chiralpak IA (0.83–4.5) Chiralpak IB (0.86–3.18)	UV	[55]
KTP, FL, PIP, TIPA	Nartardiamide DMB	CSP	(0.16–2.75)	UV	[75]
CAP, FNP, FL, IBP, INP, NPX	Chiralpak IB	CSP	(1.2–4.0)	UV	[76]
IBP, EPD, PEPD, INPN PX, CAP, SUP,	20-*O*-allyl-β-cyclodextrin, TB-β-CD	CSP	3 µM, (2.97) 11 µM, (0.76)	UV	[87]
KTP, IBP, FNP, FL	HP-β-CD	CMPA	(1.3–2.5)	UV	[78]
IBP	CalixBz-Cl	CSP	-	UV	[88]
FL	ChirobioticV	CSP	0.5 μg/mL	UV	[79]
KTP	Chirex 3005, Kromasil CHI-II	CSP	Chirex 3005 UV (1.210) Kromasil CHI-II (7.204)		[79]
IBP	α-acid glycoprotein	CSP	R: 1.16, S: 1.37	UV	[66]
IBP, CPF, NPX, KPF,	ME-/MEAC-β-CD	CSP	α = 1.0	UV	[80]
FL, INPF	Chirobiotic: V&V2	CSP	(0.00–1.57)	UV	[82]
NPX	Native fluorescent Carboxylic acid	Fluophase RP column	<11 fmol	FLD	[83]
KTP	Norvancomycin	CMPA	R: 0.86 ng S: 0.78 ng	UV	[84]
NPX	Methy-β-cyclodextrin	CMPA	(1.69)	UV	[85]
KTP, IBP	DNS-Apy	PCD	Sub-pmol	FLD	[86]

**Table 4:** Reaction performed in microreactors.

Reaction	Chip Material	Solvent	Conversion (%)	Ref.
Fluorination	Ni or Cu	Nitrogen gas	90–99	[101]
Oxidation	Al	None	75–99	[102]
Dehydration	Glass/PDMS	EtOH	85–95	[103]
Phase transfer	Glass	EtOAc	100	[104]
Aldo	Glass	Tetrahydrofruan	100	[105]
Peptide synthesis	Glass	Dimethyl formamide	100	[106]
Nitrations	Channel stainless-steel microreactor	HNO_3_	97.2	[107]
Azidations	Stainless-steel tube reactor	Tetrahydrofura	94	[108]
Fluorinations	PTFE tube reactor	Diethylamino sulfur	40–100	[109]
Grignard reactions	PTFE tube reactor	-	96	[110]
Photochemistry	Microstructured reactor	-	97	[111]
Automated reaction screening	Borosilicate glass microreactor	-	96	[112]
Esterfication, tranesterfication	Stainless-steel microchannel	Methanol/H_2_SO_4_	90, 99.5	[113]
Generation and reaction of Oxiranyllithiums	Stainless-steel T-shaped micromixer	THF	97–100	[114]
Synthesized of dimethyl ether from syngas	Stainless-steel micropacked reactor	Al_2_O_3_	80–100	[115]
Nitration of o-nitrotoluene	Stainless steel T-shaped microchannel	Sulfuric acid	94	[116]
Steam reforming of methane over Ni catalyst	Microchannel	Ni catalyst	98	[117]
